# Novel roles of phentolamine in protecting axon myelination, muscle atrophy, and functional recovery following nerve injury

**DOI:** 10.1038/s41598-022-07253-w

**Published:** 2022-02-28

**Authors:** Zarin Zainul, Bo Ma, Mert Koka, Jenny L. Wilkerson, Yuma T. Ortiz, Laura Kerosuo, Vijayendran Chandran

**Affiliations:** 1 Department of Pediatrics, College of Medicine, University of Florida, Gainesville, FL 32610 USA; 2 Department of Pharmacodynamics, College of Pharmacy, University of Florida, Gainesville, FL 32610 USA; 3 Neural Crest Development and Disease Unit, National Institute of Dental and Craniofacial Research, National Institutes of Health Intramural Research Program, Bethesda, MD 20892 USA; 4 Department of Neuroscience, College of Medicine, University of Florida, and McKnight Brain Institute, Gainesville, FL 32610 USA

**Keywords:** Neuroscience, Regeneration and repair in the nervous system

## Abstract

Incomplete functional recovery after peripheral nerve injury (PNI) often results in devastating physical disabilities in human patients. Despite improved progress in surgical and non-surgical approaches, achieving complete functional recovery following PNI remains a challenge. This study demonstrates that phentolamine may hold a significant promise in treating nerve injuries and denervation induced muscle atrophy following PNI. In a sciatic nerve crush injury mouse model, we found that phentolamine treatment enhanced motor and functional recovery, protected axon myelination, and attenuated injury-induced muscle atrophy in mice at 14 days post-injury (dpi) compared to saline treatment. In the soleus of phentolamine treated animals, we observed the downregulation of phosphorylated signal transducer and activator of transcription factor 3 (p-STAT3) as well as muscle atrophy-related genes Myogenin, muscle ring finger 1 (MuRF-1), and Forkhead box O proteins (FoxO1, FoxO3). Our results show that both nerve and muscle recovery are integral components of phentolamine treatment-induced global functional recovery in mice at 14 dpi. Moreover, phentolamine treatment improved locomotor functional recovery in the mice after spinal cord crush (SCC) injury. The fact that phentolamine is an FDA approved non-selective alpha-adrenergic blocker, clinically prescribed for oral anesthesia reversal, hypertension, and erectile dysfunction makes this drug a promising candidate for repurposing in restoring behavioral recovery following PNI and SCC injuries, axonal neuropathy, and muscle wasting disorders.

## Introduction

In humans, regrowth of damaged peripheral nerves is often incomplete, resulting in partial or complete loss of motor and sensory functions causing functional disability in the patients of PNI^1^. The major contributing factors for the incomplete functional recovery include the slower rate of axonal regeneration, disruption in axonal continuity, impaired axon myelination, and denervation of the target organs^1,2^. In treating PNI, microsurgical approaches are insufficient to address these cellular and molecular events associated with the injury. Several studies have documented the neuroprotective effects of the hormones and pharmacological compounds in achieving functional recovery after injury^3,4^. Physical, laser or cell-based therapies^5^ have been reported in improving functional recovery. Despite their positive effects on functional outcomes, none of these approaches provide a complete cure for the PNI^6^. Overall, the success in the post-injury functional recovery is far from satisfactory. Muscle denervation is also one of the contributing factors towards incomplete functional recovery. Upon denervation, the muscle undergoes atrophy through the increased expression of atrophy-related genes such as Myogenin, muscle ring finger 1 (MuRF-1), and the transcription factors (TFs) such as Forkhead boxO (FoxO1, FoxO3)^7^. The attenuation of atrophy-related gene expression may prevent muscle atrophy that could improve functional recovery after PNI. However, this aspect of improving functional recovery has not been investigated much in the past and requires further study.

We previously employed a systems approach to characterize the intrinsic gene network associated with axonal outgrowth after PNI and experimentally validated several network predictions^8^. We identified a core regeneration-associated gene network, which is induced after PNI and necessary for regeneration^8^. We reasoned that if we could identify a small molecule whose effect on gene expression in injured neurons closely approximated this core signaling network associated with regeneration, such a compound should promote neurite outgrowth and improve functional recovery after PNI. We applied this drug repurposing approach^8^ in identifying an existing small molecule that could potentially improve functional recovery in mice after peripheral nerve injury than its established utility. We identified a candidate molecule, phentolamine, an FDA-approved alpha-blocker drug for the local anesthesia reversal^9^, prescribed for hypertension, urinary retention^10^, and erectile dysfunction^11^. We designed this study to unravel and evaluate the effects of phentolamine in DRG neuronal outgrowth in vitro in the presence of growth-inhibitor and investigate its impact on the motor and behavioral recovery followed by detailed morphological analysis of sciatic nerves and muscles to evaluate overall functional recovery in a sciatic nerve injury mouse model.

## Results

### Phentolamine enhances mouse DRG neuronal outgrowth on non-permissive substrate in vitro

**Figure 1:**
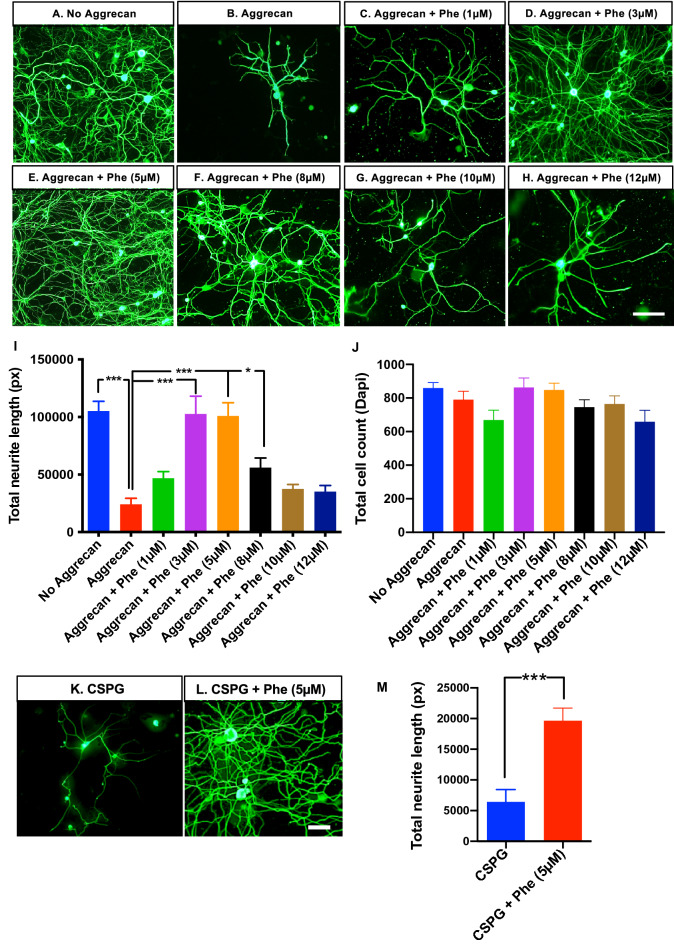
Effect of phentolamine on DRG neuron outgrowth in vitro. (**A**–**H**) Representative images of DRG neurons from 4–6 week old C57BL/6 J mice, cultured for 72 h on the mixture of poly-d-lysine PDL and laminin (Lam) substrates, (**A**) without aggrecan (**B**) with aggrecan (**C**–**H**) aggrecan with phentolamine (Phe) at concentrations, (**C**) 1 µM, (**D**) 3 µM, (**E**) 5 µM, (**F**) 8 µM, (**G**) 10 µM, and (**H**) 12 µM. Cultured neurons were immunostained with an anti-neuronal marker, β-tubulin III, to identify the neurite outgrowth in DRG neurons (green in color) and imaged with 20 × objective using Olympus microscope (scale bar, 100 µm). (**I**) Quantification of total neurite length using NeurphologyJ. (**J**) Quantification of total cell count (based on Dapi staining) using ImageJ. One-way ANOVA with Benjamini and Hochberg false discovery rate correction for multiple comparisons was performed to determine significance among the treatment conditions (*n* = 4 experiments repeated in duplicates). (**K**,**L**) Representative images of DRG neurons (72 h culture), cultured on PDL, and Lam substrates (**K**) with chondroitin sulfate proteoglycan (CSPG) (**L**) with CSPG and Phe with 5 µM concentration. (**M**) Quantification of total neurite length was done as in (**I**), and an unpaired t-test with Welch’s correction was performed to calculate statistical significance. Data are shown as Mean ± SEM (*p ≤ 0.05, **p ≤ 0.01, ***p ≤ 0.001).

We utilized the gene lists from our previously identified core regeneration-associated gene network^8^ as a signature to query a publicly available database of drug-related signature profiles known as the CREEDS^12^. We chose phentolamine from the top three matching signature patterns based on the signed Jaccard Index score^12^ for further analysis (Supplementary Fig. 1). We first tested whether mouse DRG neuronal outgrowth could be enhanced by phentolamine in the presence of axon growth inhibitory substrate, aggrecan. Mouse DRG neurons were cultured on a growth-permissive substrate containing 100 μg/mL poly-d-lysine (PDL) and 10 μg/mL laminin and served as negative controls (Fig. 1A). DRG neurons cultured in the presence of growth inhibitory substrate, aggrecan in addition to PDL, and laminin served as a positive control (Fig. 1B). The neurons with PDL, laminin, and aggrecan were treated with 1, 3, 5, 8, 10, and 12 μM concentrations of phentolamine (Fig. 1C–H), respectively, for 72 h. The aggrecan treatment significantly inhibited neuronal growth compared to neurons without aggrecan (p = 0.0001). In turn, in the presence of aggrecan, the treatment of phentolamine significantly increased the DRG neurite length at 3, 5, and 8 μM concentrations, compared to no phentolamine treatment (p = 0.0001, p = 0.0001 and p = 0.0174), respectively (Fig. 1I). The higher concentrations of phentolamine, such as 10 and 12 μM, had no significant effect on the DRG neurite length (p = 0.3238, p = 0.4051), respectively, compared to neurons with aggrecan and without phentolamine (Fig. 1I). The total number of cells plated for each culture condition was not significantly different among the groups. (Fig. 1J). To test whether DRG neurons can overcome the growth inhibition exacerbated by a mixture of chondroitin sulfate proteoglycan (CSPG), we treated the neurons with 5 μM concentration of phentolamine (Fig. 1K,L). Consistently, the total neurite length was significantly increased (p = 0.0008) when treated with 5 μM concentration of phentolamine compared with no treatment (Fig. 1M). Based on these results, phentolamine enhanced DRG neuronal outgrowth to overcome the growth inhibition caused by growth inhibitors, aggrecan, and CSPG. Next, we asked if phenoxybenzamine, another non-selective alpha-blocker, could also enhance DRG neuronal outgrowth in vitro in the presence of aggrecan, similar to phentolamine. Unexpectedly, phenoxybenzamine did not improve the DRG neuronal growth at either 5 or 10 μM concentration (p = 0.1408, p = 0.0853), respectively, compared to the neurons with aggrecan only (Supplementary Fig. 2C–E). An approximately equal number of total cells were plated in all treatment groups (Supplementary Fig. 2F). Consistently, phentolamine significantly enhanced neurite length compared to the neurons with aggrecan only (p = 0.0001) (Supplementary Fig. 2A,B,E). Unlike phentolamine, phenoxybenzamine did not affect DRG neuronal outgrowth in vitro. These observations suggest that phentolamine enhanced the DRG neuronal outgrowth in vitro through a different mechanism than its known alpha-receptor blocking activity.

### Phentolamine accelerates motor function and behavioral recovery following sciatic nerve injury

**Figure 2:**
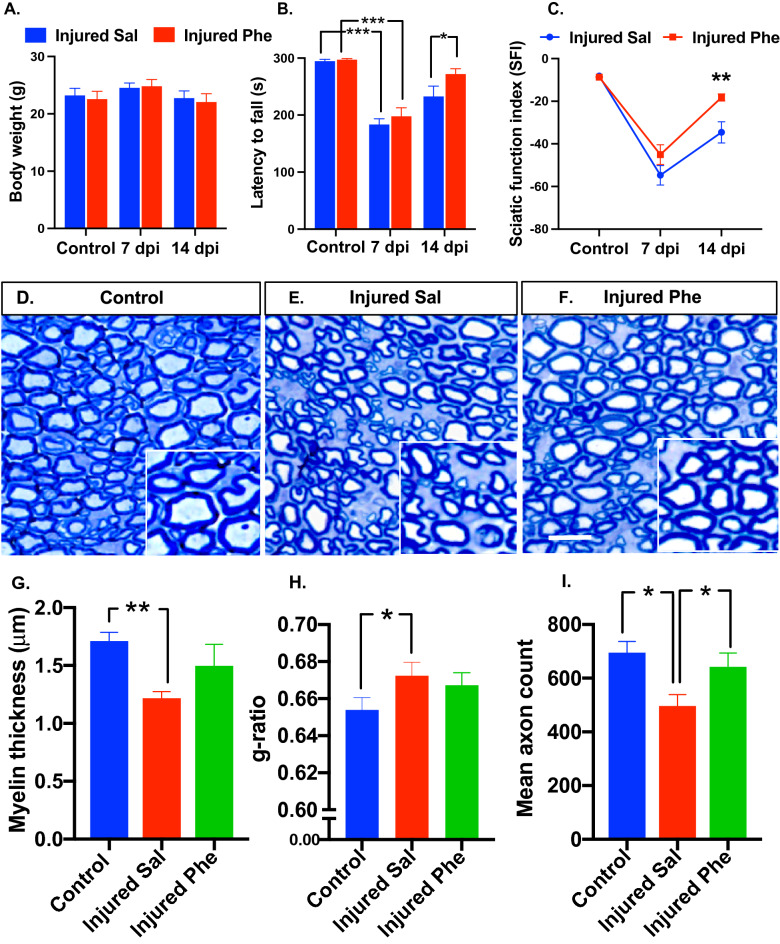
Assessment of post-injury functional recovery and sciatic nerve axon morphology. (**A**) Mice body weights in grams (*n* = 8–12 per treatment group per time point). (**B**) Rotarod test was performed to access the motor coordination and balance of the mice before, 7 and 14-day post-injury (dpi) in mice treated with saline and phentolamine. The latency until the mice fell off the rotating rod was monitored for 300 s (*n* = 8–12 per group at each time point). Two-way ANOVA with Benjamini and Hochberg false discovery rate correction for multiple comparisons was performed to determine the significance. (**C**) Sciatic function index (SFI) was evaluated by performing an inked-foot print test at pre-injury, 7, and 14 dpi in both treatment groups (*n* = 8–12 per group per time point). Two-way ANOVA with Benjamini and Hochberg false discovery rate correction for multiple comparisons was performed to determine the significance. (**D**–**F**) Transverse semithin sections of 14 dpi sciatic nerves stained with toluidine blue from control, saline, and phentolamine treated mice (scale bar, 50 µm). (**G**) Myelin sheath thickness was measured as previously done^42^. One-way ANOVA with Benjamini and Hochberg false discovery rate correction for multiple comparisons was performed to determine the significance. (**H**) Area based g-ratios were measured using an image J plugin, g-ratio calculator by analyzing of a total of 150 randomly chosen myelinated axons (excluding the axons whose myelin was touching another neighboring axons) from the sections of individual sciatic nerves per treatment group (control *n* = 3, saline *n* = 5, phentolamine *n* = 4). Kruskal–Wallis test (non-normally distributed data) followed by Benjamini and Hochberg false discovery rate correction for multiple comparisons was performed. (**I**) Quantification of the mean axon count in 200 µm^2^ area selected from the middle of the sciatic nerve section image (control *n* = 3, saline *n* = 5, phentolamine *n* = 5). One-way ANOVA followed by Benjamini and Hochberg correction was performed to calculate statistical significance. Data are shown as Mean ± SEM (*p ≤ 0.05, **p ≤ 0.01, ***p ≤ 0.001).

Since phentolamine significantly enhanced DRG neuronal outgrowth in vitro, we continued our investigation utilizing a mouse model of sciatic nerve injury. Following injury, mice were treated with saline or phentolamine for 14 days with a daily dose of 20 mg/kg through an intraperitoneal route. The body weights were not significantly different in any treatment group at any time point (Fig. 2A). We focused on examining the effects of phentolamine on motor function recovery. First, we evaluated the effect of phentolamine on mouse motor coordination and balance by performing the rotarod test on days 7 and 14 after injury. Before sciatic nerve injury and treatment, mice did not show any impairment in their rotarod performance (p = 0.8669) as both pre-assigned mice groups were able to perform on the rotarod for a maximum of 300 s (Fig. 2B). At 7 dpi, the rotarod performance of both saline and phentolamine treated mice was significantly compromised compared to their pre-injury state (p = 0.0001, p = 0.0001), respectively suggesting robust crush injury to the sciatic nerves (Fig. 2B). Following PNI at 14 dpi, phentolamine treated mice showed significantly higher rotarod performance compared to saline group (p = 0.0267) (Fig. 2B). Next, we set out to determine motor and sciatic nerve function by performing walking track analysis as assessed in the context of sciatic function index (SFI) using measurements of mice walking patterns such as footprint length, toe spread, and intermediate toe spread. Before the injury and treatment, the SFI was not different in both mice groups (p = 0.9110). At 7 dpi, the SFI was decreased in both saline and drug treated animals compared to their pre-injury state (p = 0.0001, p = 0.0001), respectively confirming the successful sciatic nerve injury. At 14 dpi, the SFI was significantly improved in the phentolamine treated animals compared to saline group (p = 0.0036) (Fig. 2C). Based on both rotarod and SFI analysis, we found that once a daily administration of phentolamine (20 mg/kg) significantly enhanced functional recovery in mice at 14 dpi following peripheral nerve injury.

### Phentolamine treatment protects myelination and the number of axons after sciatic nerve injury

Improved post-injury behavioral and motor functions are associated with the axon number and the extent of myelination. Next, we evaluated the sciatic nerve morphology at 14 dpi by performing toluidine blue staining in all treatment groups (Fig. 2D–F). Three parameters, the myelin thickness, area-based g-ratio, and the number of axons, were evaluated. The axon myelin thickness of saline-treated animals was significantly decreased (p = 0.0039) compared to control. Compared to the saline group, phentolamine treated animals had elevated myelin thickness but not statistically significant (p = 0.1033) (Fig. 2G). Consistent with this finding, we found that the average area-based axon g-ratio in saline-treated mice was significantly higher, indicating thinner myelination compared to g-ratio of control nerves (p = 0.0166). On the contrary, there was no significant difference in g-ratio between phentolamine and saline-treated mice groups (p = 0.4097) (Fig. 2H). The benefits of phentolamine treatment were also examined by analyzing the mean number of axons in the 200 um^2^ area. The mean axon count at 14 dpi was significantly lower in the saline treatment group (p = 0.0216) compared to controls. The phentolamine treated group had a significantly higher mean axon count (p = 0.0432) compared to the saline group. There were no significant differences in the mean axon count between the control and phentolamine group (p = 0.4906) (Fig. 2I). These findings suggest that phentolamine treatment may have a protective effect on myelination and the number of axons upon peripheral nerve injury.

### Phentolamine attenuates muscle atrophy after sciatic nerve injury in mice

**Figure 3:**
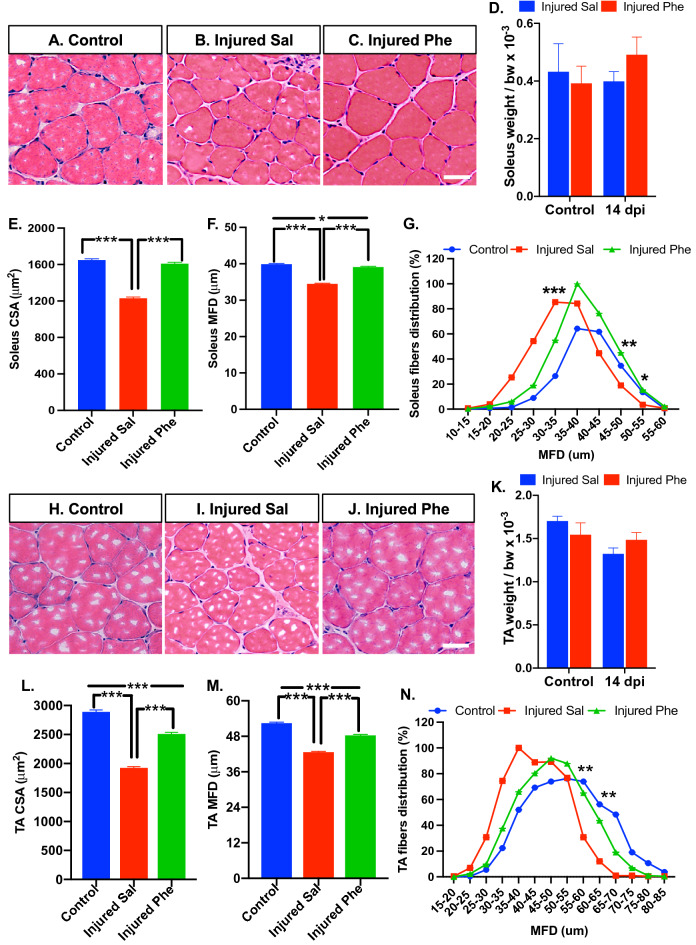
Effect of in vivo phentolamine treatment on post-injury soleus and TA muscle morphology. (**A**–**C**) Representative images of soleus muscle cross-sections, with hematoxylin–eosin (H&E) staining (scale bar, 100 µm). (**D**) Soleus muscle weigh normalized by body weight × 10^–3^ (control *n* = 5, 14 dpi *n* = 8 per treatment group). (**E**) Quantification of soleus cross-section area (CSA) (*n* = 900 fibers from 4 individual mice per treatment group). (**F**) Quantification of soleus minimal Feret’s diameter (MFD) (*n* = 900 fibers from 4 individual mice per treatment group). (**G**) Distribution of soleus muscle fibers against MFD (*n* = 4 per group). One-way ANOVA followed by Benjamini and Hochberg correction was performed to calculate statistical significance. (**H**–**J**) Representative images of TA muscle cross-sections, with hematoxylin–eosin (H&E) staining (scale bar, 100 µm). (**K**) TA muscle weigh normalized by body weight × 10^–3^ (control *n* = 5, 14 dpi *n* = 8 per treatment group). (**L**) Quantification of TA CSA (*n* = 1100 fibers from 4 individual mice per treatment group). (**M**) Quantification of TA MFD (*n* = 1100 fibers from 4 individual mice per treatment group). (**N**) Distribution of TA muscle fibers against MFD (*n* = 4 per group). One-way ANOVA followed by Benjamini and Hochberg correction was performed to calculate statistical significance. Data are shown as Mean ± SEM (*p ≤ 0.05, **p ≤ 0.01, ***p ≤ 0.001).

Sciatic nerve injury leads to the target muscle denervation that results in muscle atrophy. We examined if phentolamine treatment can improve injury-induced muscle atrophy in the soleus and tibialis anterior (TA) muscles. First, we analyzed the cross-section area (CSA) and minimal Feret’s diameter (MFD) of the H&E stained soleus muscle cross-sections obtained from the control, saline, and phentolamine treated animals at 14 dpi (Fig. 3A–C). The wet muscle weight of the soleus muscles was not significant in either treatment group before and after injury (Fig. 3D). In soleus muscle, the CSA and MFD of the saline-treated animals were significantly reduced compared to uninjured control (p = 0.0001, p = 0.0001), respectively (Fig. 3E,F). Phentolamine-treated animals showed higher CSA and MFD (p = 0.0001, p = 0.0001), respectively, compared to saline-treated animals. No significant difference was found in CSA between drug-treated and control animals (p = 0.0712), whereas MFD was still significantly reduced in the drug-treated group compared to control (p = 0.0127) (Fig. 3E,F). The evaluation of muscle fiber distribution revealed a shift towards a higher number of small-sized (20–35 μm) myofibers in saline-treated muscles, which was significant in the fibers of 30–35 μm diameter compared to controls and phentolamine treated muscles (p = 0.0002), (p = 0.0202), respectively (Fig. 3G). The saline group exhibited a lower number of larger muscle fibers, and it was significant in the myofibers of 45–50 and 50–55 um compared to the control (p = 0.0078) and phentolamine group (p = 0.0368), respectively (Fig. 3G). Next, we evaluated the TA muscle morphology in all treatment groups (Fig. 3H–J). There was no difference in the TA muscle weights before and after injury at 14 dpi (Fig. 3K). Like the soleus, TA muscle in phentolamine-treated animals showed significantly improved CSA and MFD than saline-treated mice (p = 0.0001, and p = 0.0001) (Fig. 3L,M), respectively. However, the CSA and MFD were significantly reduced in the phentolamine-treated group compared to control (p = 0.0001, p = 0.0001), respectively (Fig. 3L,M). Consistent with soleus, the distribution of TA muscle fibers in the saline group was shifted towards the higher number of smaller fibers. The number of muscle fibers of 55–60 and 60–65 μm diameter was significantly reduced in saline-treated group (p = 0.0089, p = 0.0069) compared to the control (Fig. 3N). No significant difference was observed in the fiber distribution between control and phentolamine-treated mice muscles (Fig. 3N). Together, this data suggested that the phentolamine treatment attenuated the soleus and TA muscle atrophy at 14 dpi in mice after sciatic nerve injury.

### Phentolamine modulates the expression of muscle atrophy genes

**Figure 4:**
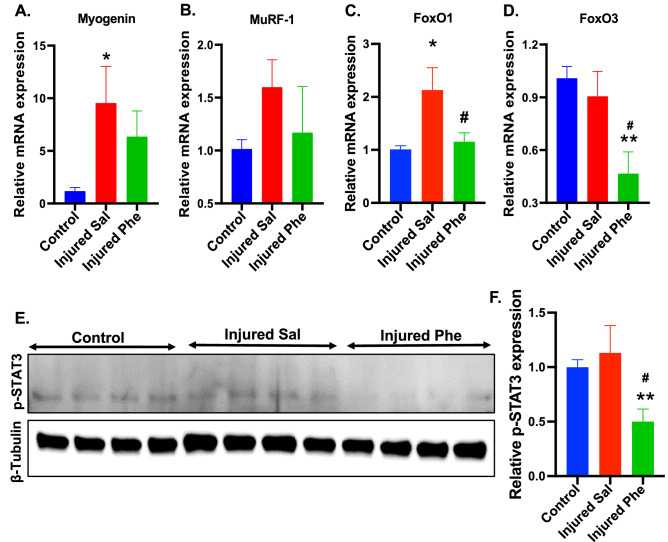
Effects of phentolamine on the muscle atrophy genes in vivo. (**A**–**D**) Relative mRNA expression of the genes (**A**) Myogenin, (**B**) MuRF-1, (**C**) FoxO1, and (**D**) FoxO3 was quantified by qPCR in the soleus at 7 dpi, normalized to HPRT-1. Statistical significance was determined using one-way ANOVA followed by Benjamini and Hochberg correction was performed to calculate statistical significance (*n* = 5 animals per treatment group). (**E**) The representative cropped immunoblot images showing the expression of phosphorylated STAT3 at Ser727 (p-STAT3) and β-tubulin in the soleus muscles at 7 dpi. After transfer, membranes were cut using standard marker and stained separately with primary antibodies (p-STAT3 and β-tubulin) followed by secondary antibodies. Original immunoblot images are provided in the Supplementary Fig. S4. (**F**) Quantification histogram of western immunoblot comparing p-STAT3 expression levels among groups (*n* = 4–5 per treatment group). One-way ANOVA followed by Benjamini and Hochberg correction was performed to calculate statistical significance. Data show the average of two-independent experiments. Data are presented as Mean ± SEM (*p ≤ 0.05, **p ≤ 0.01, ***p ≤ 0.001). ^#^p ≤ 0.05, ^##^p ≤ 0.01, ^###^p ≤ 0.001 represent significance between injured Sal and Injured Phe treatment groups.

Muscle atrophy is linked with the upregulation of genes such as Myogenin, muscle ring finger 1 (MuRF-1)^13^, and the transcription factors (TFs) such as Forkhead box (FoxO1 and FoxO3)^14^. Based on qPCR analysis at 7 dpi in the soleus muscle, the expression of Myogenin was upregulated in the saline-treated animals compared to control (p = 0.0334). There was no significant difference in the expression of Myogenin between phentolamine and control groups (Fig. 4A). The MuRF-1 expression was elevated in the saline-treated mice, though not significant compared to control and phentolamine treated animals (p = 0.1888, p = 0.3252), respectively (Fig. 4B). FoxO1 expression was significantly higher in the saline group than in the controls (p = 0.0115). In turn, FoxO1 expression in phentolamine group was significantly reduced compared to saline-treated animals (p = 0.0237) (Fig. 4C). The expression of FoxO3 was significantly reduced in the phentolamine group compared to control (p = 0.0060) and saline-treated group (p = 0.0194) (Fig. 4D). These results suggest that phentolamine significantly decreases the expression levels of atrophy-related genes and TFs in the soleus muscle at 7 dpi. We also evaluated the expression levels of atrophy-related genes in the TA muscle. The expression of Myogenin was not significantly elevated in saline and phentolamine-treated animals (p = 0.4095, p = 0.7413) compared to control (Supplementary Fig. 3A). The MuRF-1 expression was elevated in the saline-treated animals though not significant compared to control (p = 0.1070) and phentolamine-treated animals (p = 0.5989) (Supplementary Fig. 3B). The FoxO1 expression was highly elevated in the phentolamine group (p = 0.0245) but not in the saline-treated group (p = 0.1760) compared to control animals (Supplementary Fig. 3C). The expression level of FoxO3 was significantly elevated in both saline and phentolamine treatment groups (p = 0.0104 and p = 0.0312), respectively, compared to control animals (Supplementary Fig. 3D). These findings suggest that the soleus and TA muscles are differentially expressing the mRNAs of the atrophy related genes upon sciatic nerve injury.

Previous studies show that the STAT3 activation in myofibers leads to protein degradation by upregulating E3 ubiquitin ligase, MuRF-1^15,16^, and activates autophagy by inducing autophagy-related genes such as FoxO1 and FoxO3 that contribute to the muscle atrophy^16,17^. This directed us to look at the expression level of phosphorylated STAT3 (Ser727) (p-STAT3) by performing western immunoblotting in the soleus muscle tissues at 7 dpi. Surprisingly, the expression of p-STAT3 was significantly downregulated in the phentolamine-treated animals compared to saline-treated (p = 0.0092) and uninjured control animals (p = 0.095). The expression of p-STAT3 in the saline group was not significantly different from control (p = 0.5601) (Fig. 4E,F). This data strongly suggests that the phentolamine may have attenuated the muscle atrophy by suppressing the activation of the STAT3 that may have contributed to the downregulation of ubiquitin–proteasome (MuRF-1) and autophagy pathway through suppressing FoxO1 and FoxO3.

### Phentolamine enhances behavioral recovery in mice following spinal cord crush injury

**Figure 5:**
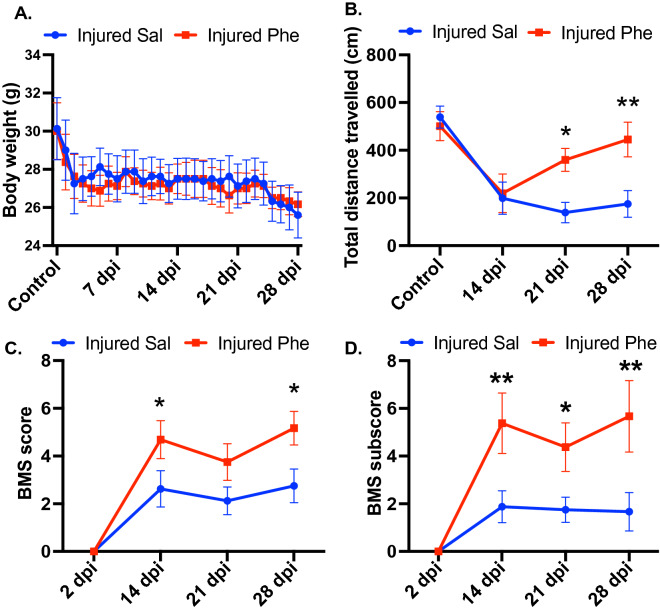
Effects of phentolamine on functional recovery after SCI. (**A**) Body weight of mice were recorded daily up to 28 days after SCI. (**B**) Open field locomotor test was performed before injury and 14, 21 and 28 dpi in mice treated with saline and phentolamine. Mice from both treatment groups were allowed to walk freely in an open field and the total travelled distance within the 20 min time period is calculated in centimeters (cm.) by the software Versamax. (**C**) BMS scores and (**D**) BMS subscores were evaluated at 2, 14, 21 and 28 days post SCC injury. The average BMS scores of the left and right hind limbs (**C**) and the sum of subscores (**D**) were calculated for each mouse. Two-way ANOVA with Benjamini and Hochberg false discovery rate correction for multiple comparisons was performed to determine the significance. N = 6–8 animals per treatment group. Data are shown as Mean ± SEM (*p ≤ 0.05, **p ≤ 0.01, ***p ≤ 0.001).

Since phentolamine treatment significantly improved functional recovery after peripheral nerve injury, we sought to determine whether phentolamine enhances behavioral recovery after central nervous system (CNS) injury. To examine this, we performed spinal cord crush (SCC) injury in mice and treated them with saline and phentolamine until 28 days post-injury. After the SCC, as expected, we observed a drop in body weight in both groups. However, we did not observe any significant body weight difference between saline and phentolamine-treated animals up to 28 days after SCC (Fig. 5A). To evaluate the effects of phentolamine on spontaneous locomotion and behavioral activity after SCC injury, we performed an open-field locomotor test. Because of SCC, the mice in both treatment groups at 14 days post-SCC displayed reduced locomotor activity (Fig. 5B). However, the total distance traveled by the phentolamine-treated mice was significantly higher compared to saline-treated animals at 21 (p = 0.0106) and 28 days post-SCC (p = 0.0069) (Fig. 5B). Next, we performed a Basso mouse scale (BMS) open-field locomotion test that evaluates locomotor functions such as paralysis, weight support, and stepping pattern^18^. The BMS score of phentolamine-treated animals was significantly improved at 14 and 28 dpi compared to saline-treated mice (p = 0.0184, p = 0.0169 (Fig. 5C). Next, we examined the BMS subscores, which assess finer aspects of locomotion that do not follow a typical pattern of recovery and are not reflected in a change in the overall score on the BMS^18^. Evaluation of BMS subscores revealed significantly higher subscores on 14, 21, and 28 days after injury in phentolamine treated animals compared to saline treatment (p = 0.0036, p = 0.0265, p = 0.0040), respectively (Fig. 5D). Overall, these results suggest that phentolamine improved behavioral recovery after both PNS and CNS injury in mice.

## Discussion

Peripheral nerve trauma often results in incomplete functional recovery. Despite the latest improvements in treating peripheral nerve injuries, achieving a satisfactory functional recovery level is an unmet challenge. In the current study, we sought to unravel the roles of phentolamine, whether it could restore the functional outcomes after sciatic nerve injury in the mouse model of peripheral nerve injury. If yes, what are the contributing factors and mechanisms? Phentolamine is a non-selective alpha-blocker used to treat patients with hypertension^10^, erectile dysfunction^11^, and pheochromocytoma^19^. Most of the alpha-1 receptors are present on the vascular smooth muscles and activated by the release of epinephrine and norepinephrine (NE) resulting in vasoconstriction. Alpha-2 receptors are mainly located on the peripheral nerve endings and inhibit the release of NE. Blocking the alpha-1 and alpha-2 receptors with the non-selective alpha-blockers results in vasodilation^19^. We found that the treatment of phentolamine robustly enhanced DRG neuronal outgrowth in vitro in the presence of growth inhibitors, aggrecan, and CSPG. Next, we were curious if the non-selective alpha-blocker such as phenoxybenzamine has similar effects on DRG neuronal growth as we observed with the phentolamine. Unexpectedly, phenoxybenzamine did not enhance DRG neural growth in the presence of aggrecan when tested with two different concentrations, such as 5 and 10 μM. We also looked at the effects of an alpha-agonist, oxymetazoline, on the DRG neuronal growth. However, we did not observe any opposite effects of an alpha-agonist compared to phentolamine, which further emphasized that phentolamine treatment enhancing DRG neuronal growth in vitro is unlikely through its alpha-receptor blocking property.

In the mouse model of PNI, we found that a daily dose of 20 mg/kg for up to two weeks in mice enhanced the functional recovery at 14 dpi, as shown by rotarod and the SFI results. Functional recovery primarily depends on the number of axons, the extent of myelination, and axon regeneration, whereas the recovery of denervation induced muscle atrophy is secondary^20^. Consistent with rotarod and SFI results, the number of axons and myelination were improved at 14 dpi after phentolamine treatment. The significant motor and functional improvements are associated with the nerve conduction velocity. However, we do not know whether phentolamine affected the nerve conduction in injured sciatic nerves contributing to the improved SFI. However, a protective effect on myelination and higher axon numbers may explain an improved functional recovery, evaluated by SFI. A selective alpha-blocker, prazosin, has been shown to improve the nerve conduction in streptozocin-induced diabetic neuropathic rats by increasing vasa nervorum perfusion, indicating the importance of vasodilation in relieving the neuropathic symptoms^21^, which are often associated with diminished neuronal regeneration. Phentolamine has been utilized extensively as a sympatholytic in human studies^22–24^. Failing to achieve functional recovery is also attributed to the denervation induced muscle atrophy^25,26^. We show, at 14 dpi, the muscle cross-section area and diameter were significantly increased in phentolamine treated animals compared to saline treatment. It is known that muscle atrophy genes are elevated and peak at 7 dpi after denervation^27,28^. Myogenin is upregulated in the muscles upon denervation that regulates other atrophy-related genes^29^ as MuRF-1 and contributes to the progression of atrophy. Moreover, the transcription factors, FoxO1, and FoxO3 also contribute to muscle atrophy^30,31^. In our study, we show that the phentolamine treatment significantly downregulated the muscle atrophy-related genes at 7 dpi. These findings were consistent with the muscle histology data at 14 dpi where muscle atrophy was attenuated in the phentolamine group. In turn, there was no significant difference in the expression levels of Myogenin and MuRF-1 in the TA muscle of either treatment group at 7 dpi. The expression of FoxO1 and FoxO3 in TA has not normalized at 7 dpi in the phentolamine-treated animals compared to saline. However, the TA muscle showed significantly improved CSA, and the MFD 14 dpi in phentolamine-treated animals compared to saline or control animals. STAT3 activation in myofibers leads to protein degradation by upregulating E3 ubiquitin ligase, MuRF-1^15,16^, and activates autophagy by inducing autophagy-related genes such as FoxO1 and FoxO3 that contribute to the muscle atrophy upon denervation^16,17^. This evidence directed us to look at the expression of STAT3 in the muscle at 7 dpi. We show that the activated p-STAT3 expression was significantly decreased in the phentolamine-treated group. We experimentally demonstrated the muscle-specific suppression of STAT3 upon phentolamine treatment that may have contributed to the downregulation of muscle atrophy-related genes and TFs and improved the muscle atrophy. As a validation of muscle function, further muscle electrophysiological measurements would help determine whether the phentolamine induced recovered muscles are functional. FoxO3a has been shown to play an essential role in peripheral neuronal regeneration^32^. FoxO3a expression was decreased in differentiating Schwann cells (SCs) in vivo after PNI and in cAMP-induced differentiated primary SCs^33^. The reduced level of FoxO3a and Cip/Kip family of cyclin-dependent kinase inhibitors, p27kip1, were predominant in the regenerating DRG neurons and glial cells that were largely proliferated after sciatic nerve injury^32^. Our study has shown the phentolamine-induced beneficial effects of decreased expression of FoxO1 and FoxO3 genes in the attenuation of muscle atrophy. We showed that phentolamine treatment enhanced functional recovery, attributed to the potential myeloprotection and recovered muscle atrophy post sciatic nerve injury.

Unlike the PNS, CNS axons do not grow sufficiently and cause incomplete functional recovery after injury^34^. To date, there is no treatment available to treat CNS injury and restore lost functional recovery^35^. Based on the phentolamine treatment associated results in the PNS model, we wanted to replicate these results using a different cohort of animals utilizing the SCC injury mouse model. Phentolamine-treated animals showed an improved movement evaluated by open field test starting from 21 dpi after SCC injury. In a clinical setting, an open field-related measure of total distance traveled mirrors the six-minute walk test as a clinical trial outcome in human patients^36^. The BMS score test, which evaluates the differences in the hind limb locomotor recovery was also improved in the phentolamine treated animals at 14 and 28 dpi after SCC injury. Furthermore, the BMS subscore, which is better for discriminating the differences in the fine details of the locomotion that may not be picked up by the BMS score itself^18^ showed an elevated recovery starting from 14 to 28 dpi.

There are limitations to our study. We have not evaluated the effects of different dose concentrations in vivo. For example, a dose higher than 20 mg/kg might have resulted in an earlier or complete functional recovery than what we observed at 14 dpi. Moreover, it would be interesting in the future to study the effects of phentolamine in permanent denervation mouse models. The drug delivery approaches such as transdermal^37^ or localized slow-release^4^ could also be considered in phentolamine treatment to sustain drug availability in circulating blood to minimize multiple drug dosing to the animals.

In summary, we provided the first evidence that phentolamine treatment enhanced functional recovery, protected axon myelination and attenuated muscle atrophy after PNS nerve injury. We identified that the soleus of phentolamine-treated animals has decreased expression of p-STAT3 and muscle atrophy-related genes that may have contributed to improving muscle phenotype. Interestingly, phentolamine also improved functional locomotor recovery after spinal cord crush injury in mice. Further longitudinal studies following phentolamine treatment are required to explore the axon and muscle-specific mechanisms to restore functional recovery after PNS and CNS nerve injuries. Overall, based on the obtained results, phentolamine may serve as a potential therapy in restoring injury-related functional recovery, neuropathy, and muscle wasting disorders.

## Materials and methods

### DRG neuronal cell-culture and quantification

Based on the protocol described previously^8^. 4–6-week-old C57BL/6J mice dissociated DRG neurons were plated onto the poly-D-lysine (100 µg/mL), laminin (10 µg/mL) and the aggrecan (50 µg/mL) (P-6407, L-2020, A-1960, Sigma) coated coverslips in the 24-well culture plates and cultured in Neurobasal medium (1088802; Thermo Fisher Scientific) with B27 supplement containing penicillin, streptomycin, 1 mM l-glutamine, 50 ng/mL NGF, 2 ng/mL GDNF, and 10 mM AraC at 37°. For drug treatment, DRG neurons were cultured for 72 h in the presence of the drug phentolamine at 1, 3, 5, 8, 10, 12, 20 μm concentrations. The cells were post-fixed 4% paraformaldehyde (PFA) followed by phosphate buffer saline (PBS) washing and immunostained with anti-mouse b-III-tubulin (1:1000; 801201, BioLegend). Coverslips were then inverted and mounted on the glass-slides using Prolong Gold antifade reagent with DAPI (P36935, Fisher Scientific). Images were captured covering the entire coverslip area at × 20 magnification using an Olympus Fluorescent Microscope. Total neurite length was quantified with an ImageJ plug-in, NeurphologyJ^38^ (RRID: SCR_003070). Neurphology J operates on the entire image and quantifies the neurite length in pixels. Data were obtained from at least 4 separate experiments repeated in duplicates.

### Sciatic nerve crush injury and phentolamine treatment

The sciatic nerve crush injury was performed on C57BL/6J and Thy1-YFP (B6.Cg-Tg(Thy1-YFP)16JRS/J) mice from Jackson laboratory, USA as described previously^2^. All animal experiments were carried out in accordance with relevant guidelines and regulations, and upon the approval of an Institutional Animal Care and Use Committee (IACUC) at the University of Florida (protocol number # 201709975) and in compliance with the ARRIVE guidelines. The 8- to 12-week old mice were anesthetized by injecting Ketamine (120 mg/kg)/Xylazine (16 mg/kg) intraperitoneally. An appropriate level of anesthesia was determined by toe-pinch method and an eye ointment was applied to both eyes to prevent it from dryness during the surgical procedure. After hair clipping and aseptic animal preparation, an incision was made in the skin and mid-thigh muscle of the left hind left limb under a microscope to expose the sciatic nerve, which was then carefully lifted with a glass loop, crushed for 20 s with the tip of 0.5 mm with modest and constant pressure using No. 5 Jeweler’s forceps, and then returned to its position. The skin incision was closed by suturing with a 5–0 absorbable Vicryl suture (Johnson&Johnson). The right contralateral limb and nerve served as controls. After the wound was closed, pain medication, buprenorphine (0.1 mg/kg) was administered subcutaneously and continued for three days at 12 h interval. Phentolamine hydrochloride (P7547, Sigma-Aldrich) 20 mg/kg) or saline was delivered to the mice through an intraperitoneal injection once per day starting immediately after sciatic nerve crush injury and continued for 14 days. For the reversal of xylazine, atipamezole hydrochloride (0.01 mL/gm body weight) was administered subcutaneously. Body temperature of the mice was maintained throughout the surgical and recovery period using heating pad. Randomly selected mice were pre-assigned for saline and phentolamine treatments. After the behavioral tests, mice were sacrificed at each specified time point, and the samples were collected.

### Accelerating rotarod

Mice were assessed for sensorimotor coordination before-and-after sciatic nerve injury utilizing the Rotarod apparatus (Stoelting, UgoBasile) consists of a rotating rod of at the height of 20 cm from the floor. Mice were on rod and habituated to the rod for 5 min. After 5 min, rotation was started at a speed of 4 rpm and accelerated to 40 rpm in 240 s. The latency period until the mice fell off the apparatus was monitored for 300 s. Mice were subjected to three different tests at 5 min intervals, and the maximum seconds of performance were chosen out of three values for the data analysis. Experiments were performed in the presence of two observers.

### Walking track analysis

Sciatic function index (SFI) was evaluated as previously described^2,39–41^. Mice hind paws were painted using non-toxic ink, and footprints were obtained on the absorbent side of the surface protector paper (VWR) by allowing the mice to walk along the 5 × 42 cm open corridor toward the dark ending. Clear and readable prints were chosen for analysis by measuring their paw length (PL), toe spread (TS) and intermediate toe spread (ITS) (distance between second to the fourth toe) from both legs and SFI was calculated using the previously described formula^40^. SFI values between − 10 to + 10 were considered normal, while a score of -100 reflected as total impairment. SFI was performed in the presence of two observer.

### Axon morphometric analysis

Morphometric evaluation of the sciatic nerves was done as described earlier^2^. At 14-day post-injury, crushed and non-crushed contralateral control nerves from 5 mm distal to the crush site, were collected and fixed in 1% glutaraldehyde, 4% PFA/0.1 M phosphate buffer, pH 7.3 followed by post-fixation with 1% osmium tetroxide, dehydrated in acetone, and embedded in Epon LX112 (Lass Research Industries). Semithin nerve transverse sections were stained with toluidine blue and imaged with the Olympus, Tokyo, Japan microscope using × 40 magnification. A total of 150 randomly selected individual axons per treatment groups were evaluated for myelin thickness ([Fiber diameter − axon diameter]/2)^42^, g-ratio (total axon fiber area divided by the fiber area) by utilizing an Image J Plugin (G-ratio calculator ImageJ, RRID: SCR_015580). The mean number of axons were counted in the area of 200 μm^2^ per mouse per treatment group utilizing ImageJ software (National Institutes of Health; ImageJ, RRID: SCR_003070).

### Muscle histology and quantification

Excised muscles were quickly embedded in the Tissue-Tek Cryomolds using the OCT compound (4565, 62550-01; Sakura Finetek, USA) and immediately frozen in liquid nitrogen cooled 2-methyl butane (035514, Fisher) and stored at -80 degree until sectioning. Tibialis anterior (TA) and soleus muscles sections of 7-μm thickness were cut by a Cryomicrotome (Leica CM 3050 S, Leica Microsystems), and stained with the hematoxylin/eosin (H&E) eosin. Nonoverlapping images covering the entire muscle were captured at × 40 magnification with the Olympus, Tokyo, Japan microscope. For quantifying muscle cross-section area (CSA) and the Feret’s diameter, 900 and 1100 individual muscle fibers were evaluated from the soleus and TA muscles, respectively, with the help of ImageJ (RRID: SCR_003070) software.

### Quantitative real-time polymerase chain reaction

Total RNA was extracted from soleus and TA muscles by using miRNeasy Mini Kit (217004, Qiagen). Complementary DNA (cDNA) was synthesized using Invitrogen SuperScript VILO Master Mix (11755050, Life technology). All primers were synthesized by Eurofins Genomics, USA. 1–2 μg of cDNA was used as the template for real-time polymerase chain reaction (RT PCR) analysis. Quantitative PCR reactions were performed with SYBR Green Fast Mix in a CFX Real-Time PCR machine, Bio-Rad. Changes in the expressions of Myogenin, MuRF-1, FoxO1 and FoxO3 were measured, and the data were normalized with the hypoxanthine guanine phosphoribosyl transferase1 (HPRT1). A list of all the primers with their sequences is listed in the following Table 1.

**Table 1:** Primer sequences for qRT-PCR.

Name	Primer Sequences
HPRT1 Forward	5’- AGATGGGAGGCCATCACATTGTA -3’
HPRT1 Reverse	5’- AATCCAGCAGGTCAGCAAAGAA -3’
FoxO1 Forward	5’-GCT GGG TGT TCA GGC TAA GAG-3’
FoxO1 Reverse	5’-GAG GGG TGA AGG GCA TCT-3’
FoxO3 Forward	5’-CGC TGT GTG CCC TAC TTC A-3’
FoxO3 Reverse	5’-CCC GTG CCT TCA TTC TGA-3’
Myogenin Forward	5’- CGATCTCCGCTACAGAGGC -3’
Myogenin Reverse	5’- GTTGGGACCGAACTCCAGT -3’
MuRF-1 Forward	5’-GCT GGT GGA AAA CAT CAT TGA CAT-3’
MuRF-1 Reverse	5’-CAT CGG GTG GCT GCC TTT-3’

### Western immunoblotting

The soleus muscles were homogenized in radioimmunoprecipitation assay (RIPA) lysis buffer (cat. no. J60645, Alfa Aesar by Thermo Fisher Scientific) containing 1% cocktails of protease and phosphatase inhibitor (cat. no. P8340, P0044, Sigma-Aldrich). For the estimation of protein concentration, BCA protein Assay kit (cat. no. 23225, Thermo Fisher Scientific) was used. Per lane, an equal amount of total protein was subjected to SDS-PAGE using precast 4–20% Tris glycine gels (Mini-PROTEAN TGX, cat. no. 456–1096, Bio-Rad), and transferred onto PVDF (Millipore Corp), which were blocked with 5% nonfat dry milk in Tris-buffer saline (TBS) followed by incubation with primary antibodies: rabbit monoclonal anti-phosphoSTAT3 (Ser727) (1:500; cat. no. 94994S, Cell Signaling Technology (CST)), and rabbit polyclonal anti-tubulin (1:10,000, cat no. ab6046, Abcam) at + 4° overnight. Membranes were then treated with appropriate horseradish peroxidase-coupled secondary antibody. Protein expression bands were detected by using ChemiDoc MP (Bio-Rad) Imaging System. The immunoblots were then quantified by using densitometry, an ImageJ software (NIH). The data were expressed as relative band intensities normalized to equal loading control.

### Spinal cord crush injury and phentolamine treatment

All animal experiments were carried out in accordance with relevant guidelines and regulations, and upon the approval of an Institutional Animal Care and Use Committee (IACUC) at the University of Florida (protocol number # 201709975) and in compliance with the ARRIVE guidelines. Spinal cord injury was performed on 8–12 weeks old C56BL/6 mice. As a pre-operative pain medication, buprenorphine with meloxicam and enrofloxacin were given to mice subcutaneously 10–15 min before surgery. Animals were anesthetized using isoflurane gas anesthesia. An appropriate level of anesthesia was ensured by eliciting no response to the paw-pinch. Once the mice achieved a surgical level of anesthesia, an ophthalmic ointment was applied to both eyes to prevent dryness. The body surface area of approximately 1.0 am wide and 1.5 cm long was shaved midline on the back of the mouse, followed by skin disinfection. A longitudinal incision of 1.5 cm was made midline with a scalpel blade on the back to expose the 9^th^ to 11^th^ thoracic vertebral laminae, followed by the opening of three top muscle layers. For the spinal cord crush injury, the crush was made at the T10 level of a single vertebra by using No. 5 Dumont forceps with a tip width of 0.5 mm to completely compress the entire spinal cord laterally from both sides for 5 s. The incision was closed using clips. As a post-operative care, mice received analgesics after surgery and every 12 h for three days and thereafter if mice showed signs of pain. After the surgery, mice were given 20 mg/kg phentolamine drug via intraperitoneal route for up to 28 days. Throughout the procedure, body temperature was maintained by a temperature-regulated heating pad. Two days after injury, all mice were evaluated in open field and all animals exhibiting any hindlimb movements were not studied further. Mice that passed this inclusion criterion were randomized into experimental groups for further treatments and were thereafter evaluated blind to their experimental conditions.

### Basso mouse scale (BMS) open-field locomotor test

To assess the hindlimb locomotor recovery before and after spinal cord injury in saline and phentolamine treated animals, BMS locomotor score test was performed as previously published methodology^18^. Mice were placed into the chamber and allowed to move freely for few minutes to acclimate with the chamber 4 × 4 × 12 inches dimension. The BMS score sheet included seven locomotor categories for early (ankle movement), intermediate (plantar placement, stepping), and late (coordination, paw position, trunk instability, tail) phases of recovery. The mice locomotor recovery was observed visually over a four-minute time period by an investigator blinded to the treatment conditions. BMS subscores were collected to reveal detailed characteristics about fine locomotor control such as stepping frequency, coordination, paw position, trunk stability, and tail position. BMS scores of the hind limbs and the subscores were entered on the score sheet accordingly and calculated as a sum for each mouse (Basso et al.). After the end of each mouse session, chamber surfaces were wiped and cleaned using 70% ethanol before placing the new mouse into the chamber for the next session.

### Open field locomotor test

Open field activity system was used to assess the spontaneous locomotor activity of the mice was assessed before the injury and 7, 21, 28 days post spinal cord crush injury in saline and phentolamine treated mice. Open-field testing was performed in an open field plexiglass chamber of 27.5 cm × 27.5 cm dimension with a video monitoring system that recorded mice movements within the chamber for 20 min. Each animal was placed in a chamber to acclimate for 10 min. After the initial 10 min, the recording system was turned on. A computer software (Versamax) connected to the chamber tracked and analyzed the animal locomotion during the testing. Each tested mouse was then placed in a different quadrant of the open-field chamber, and their activity was recorded for 20 min. After the recordings, mice were placed back in their home cages. After each session, chambers were cleaned and wiped with 70% ethanol before the new recording session was started. All methods, used in this study were carried out in compliance with the relevant guidelines and regulations.

### Statistical analyses

The normality of the data distribution was evaluated by using Kolmogorov–Smirnov, Shapiro–Wilk or D’Agostino-Pearson, depending on the data size. When the data is distributed normally, we have utilized ANOVA for all datasets (most cases), and in the case where the data is not distributed normally; we have used other appropriate test (individually indicated in each figure legend) since ANOVA is not advisable for the data when the data is not distributed normally. For the grouped data one or two-way ANOVA with appropriate post hoc multiple comparison test was utilized. All data were analyzed with GraphPad Prism 9 software (GraphPad Prism, RRID: SCR_002798). A p-value ≤ 0.05 was considered statistically significant, and values were reported as follows: *p ≤ 0.05, **p ≤ 0.01**, and ***p ≤ 0.001. Data are represented as means ± SEM.

## Supplementary Information

Supplementary Figures.
