# Supplementary material for: Novel roles of phentolamine in protecting axon myelination, muscle atrophy, and functional recovery following nerve injury
Source: Sci Rep. 2022 Feb 28;12:3344. doi: 10.1038/s41598-022-07253-w (PMC8885794; doi:10.1038/s41598-022-07253-w)
Supplement: Supplementary file 1 — Supplementary Figures. [file 41598_2022_7253_MOESM1_ESM.docx]

**Novel roles of phentolamine in protecting axon myelination, muscle atrophy, and functional recovery following nerve injury**

Zarin Zainul^a^, Bo Ma^a^, Mert Koka^a^, Jenny L. Wilkerson^b^, Yuma T. Ortiz^b^, Laura Kerosuo^c^, and Vijayendran Chandran^a, d, 1^

^a^ Department of Pediatrics, College of Medicine, University of Florida, Gainesville, Florida, 32610, USA.

^b^ Department of Pharmacodynamics, College of Pharmacy, University of Florida, Gainesville, Florida, 32610, USA.

^c^ Neural Crest Development and Disease Unit, National Institute of Dental and Craniofacial Research, National Institutes of Health Intramural Research Program, Bethesda, Maryland, 20892, USA.

^d^ Department of Neuroscience, College of Medicine, University of Florida, and McKnight Brain Institute, Gainesville, Florida, 32610, USA.

^1^ To whom correspondence should be addressed. Email: [vijayendran@ufl.edu](mailto:vijayendran@ufl.edu)

**
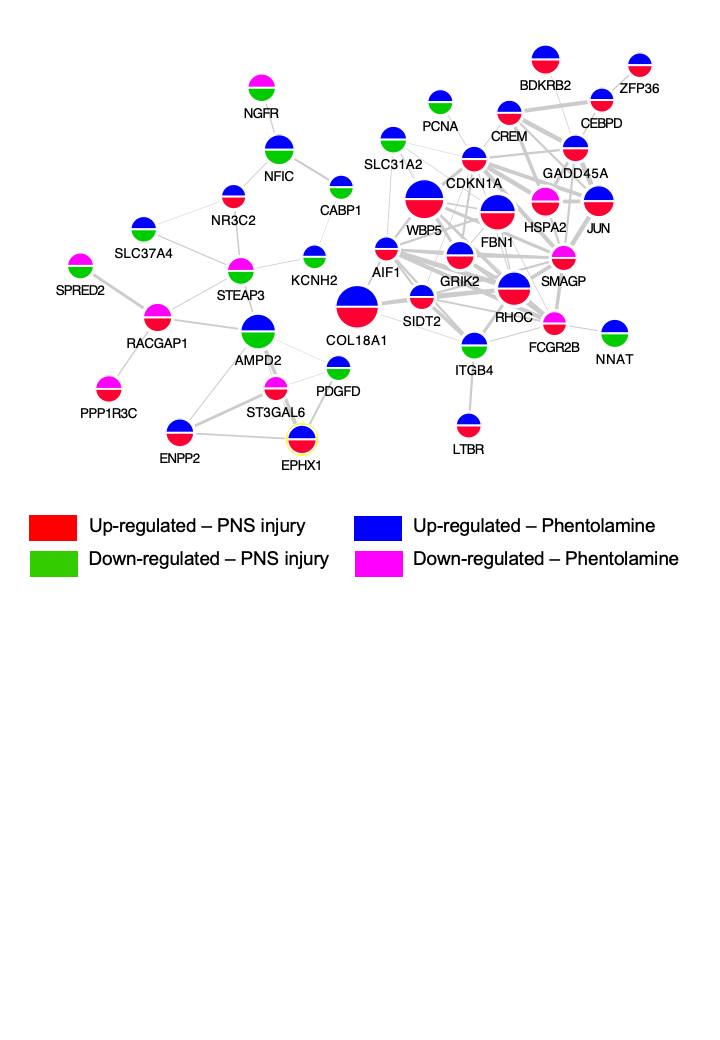
**

**Supplementary Figure 1.**

**
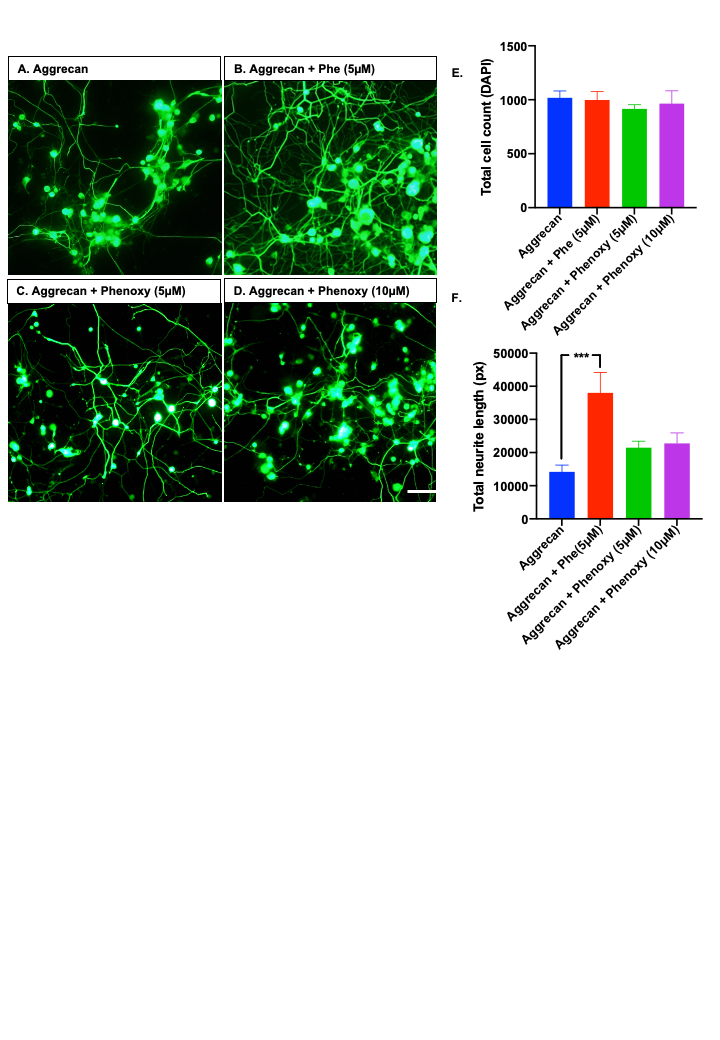
**

**Supplementary Figure 2.**

**A.**

**B.**

**C.**

**D.**

**Supplementary Figure 3.**

1-4 (Control), 5-8 (Injured Sal), 9-12 (Injured Phe)


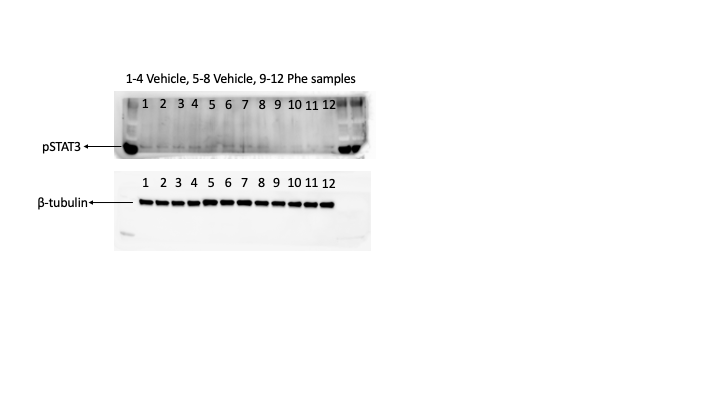


**Supplementary Figure 4.**

**Supplementary Figure legends**

**Supplementary Figure 1. Targeting regeneration associated regulatory gene network using small molecules.** Gene expression signatures after PNS injury were used to query drug-related expression signatures in the CREEDS database. Using the signature search engine within the CREEDS database, we selected phentolamine based on the signed Jaccard Index score. Correlation (edges) network of co-expressed and differentially expressed genes (nodes) after PNS injury is shown. Nodes correspond to genes and edges to a significant correlation (>0.8). Larger nodes correspond to the extent of differential expression due to phentolamine treatment. Upregulation (red) and downregulation (green) after SN lesion; upregulation (blue) and downregulation (purple) after phentolamine treatment (from CREEDS database).

**Supplementary Figure 2. Effects of phenoxybenzamine in DRG neuron outgrowth *in-vitro*.** (A-D) Representative images of DRG neurons from 4-6-week-old mice (72 hours culture) on the mixture of poly-d-lysine (PDL)) and laminin (Lam) substrates. (A) With aggrecan, (B) aggrecan plus phentolamine (5 µM), (C) aggrecan with phenoxybenzamine (Phenoxy) (5 µM) and (D) and aggrecan with phenoxy with (10 µM) concentrations. (A-D) Cultured neurons were immunostained with an anti-neuronal marker, β-tubulin III, to identify the neurite outgrowth in DRG neurons (green in color) and imaged with an Olympus microscope with 20x objective (scale bar 100 µm). (E) Quantification of total DRG neurite length by using an Image J Plugin, NeurphologyJ. (F) Quantification of the total cell count (based on Dapi positive staining). Data are shown as Mean ± SEM (*p ≤ 0.05, **p ≤ 0.01, ***p ≤ 0.001). One-way ANOVA with Benjamini and Hochberg false discovery rate correction for multiple comparisons was performed to determine significance among the treatment conditions.

**Supplementary Figure 3. Effect of phentolamine on the expression of muscle atrophy-related genes and transcription factors by qPCR in TA muscle.** (A-D) Relative mRNA expression of the genes (A) Myogenin, (B) MuRF-1, (C) FoxO1, and (D) FoxO3 was quantified by qPCR in TA muscle at 7 dpi, normalized to HPRT-1, respectively. Statistical significance was evaluated by utilizing one-way ANOVA with Benjamini and Hochberg false discovery rate correction for multiple comparisons. *N* = 5 per treatment group. Data are shown as Mean ± SEM (*p ≤ 0.05, **p ≤ 0.01, ***p ≤ 0.001). Two independent experiments were performed to replicate the results.

**Supplementary Figure 4.** Original western immunoblots showing the expression of phosphorylated STAT3 at Ser727 (p-STAT3) and β-tubulin in the soleus of control (1-4), injured saline (5-8) and injured phentolamine treated (9-12) at 7 dpi. After transfer, membranes were cut using standard marker and stained separately with primary antibodies (p-STAT3 and β-tubulin) followed by appropriate secondary antibodies. Protein expression bands were detected by using ChemiDoc MP (Bio-Rad) Imaging System.
